# Pharmacist-led academic detailing improves statin therapy prescribing for Malaysian patients with type 2 diabetes: Quasi-experimental design

**DOI:** 10.1371/journal.pone.0220458

**Published:** 2019-09-19

**Authors:** Mohamed Hassan Elnaem, Mohamad Haniki Nik Mohamed, Hasniza Zaman Huri

**Affiliations:** 1 Department of Pharmacy Practice, Faculty of Pharmacy, International Islamic University Malaysia, Kuantan, Pahang, Malaysia; 2 Department of Pharmacy, Faculty of Medicine, University of Malaya, Kuala Lumpur, Malaysia; The University of Sydney School of Pharmacy, AUSTRALIA

## Abstract

**Objective:**

Previous reports have highlighted the suboptimal utilization and prescription of statin therapy among patients with type 2 diabetes mellitus (T2DM) in the Malaysian clinical practice. This study aims to test the impact of a pharmacist-led academic detailing program on improving the overall statin therapy prescribing in Malaysian hospital and primary care settings.

**Methods:**

As a quasi-experimental design with a control group and pre-tests., we examined 1,598 medical records of T2DM subjects in six healthcare facilities in the state of Pahang, Malaysia. In all study sites, there was a pre and post-intervention assessment of the percentage of appropriate statin therapy prescribing that complied with the clinical guidelines with no potential safety issues. The intervention was an academic detailing program offered to the health care providers in three study sites, while the other three sites served as the control group. A comparison of the overall percentage of appropriate statin therapy prescribing before and after the academic detailing was performed in all intervention and control sites.

**Results:**

Overall, 797 medical records were examined in the pre-intervention phase, and 801 records were evaluated in the post-intervention phase. The academic detailing program was associated with a statistically significant difference in the proportion of appropriate statin therapy prescribing between the post-intervention phase compared to the pre-intervention phase (n = 246, 61.7% versus n = 188, 47.1%), p = 0.001. Whereas, the appropriate statin therapy prescribing in the control study sites experienced a modest change from 53.8% (214/398) to 56.7% (228/402), p = 0.220. The academic detailing showed significant increases in the proportions of appropriate statin therapy prescribing in both hospital and primary care settings.

**Conclusions:**

The academic detailing program was found to be significantly associated with a positive impact on the overall statin therapy prescribing among patients with T2DM in Malaysian hospital and primary care settings.

## Introduction

The appropriate use of lipid-lowering therapy (LLT), e.g. statin therapy has received increasing recognition and attention given the treatment gap and undertreatment (18.3% & 35%) of eligible patients with type 2 diabetes mellitus (T2DM) contradictory to the clinical guidelines recommendations in the United Kingdom and Malaysia, respectively [1,2]. Furthermore, it has been reported in a study conducted in the United States that even after reading the clinical guidelines, healthcare providers could still have gaps in their knowledge regarding the rationale and importance of the guidelines recommendations [3]. Moreover, a nationwide Indian study has highlighted that there are inconsistencies between different healthcare providers in their prescribing practice of LLT that needs to be addressed through additional educational initiatives and continuous medical education programs [4]. Consequently, promoting compliance with guideline recommendations could help in avoiding potentially preventable chronic CVD through assuring the effective, safe, and rational use of LLT [5].

In a study by Kuiper et al., to investigate the use of LLT among the high CVD risk population in the Netherlands [6], it has been concluded that there are opportunities for quality improvement interventions to improve the practice related to recommended therapeutic approaches of CVD prevention. Furthermore, previous research highlighted that pharmacists could play an important role in optimizing the use of LLT among high CVD risk subjects through reviewing the current treatment strategies and promoting the usage of high-intensity statin treatment and ezetimibe-statin combination therapy to enhance the attainment of LDL-c target values [7,8]. In a previous US study that aimed at evaluating the potential impact of pharmacists’ intervention on statin therapy, it has been found that overall 35% of pharmacists’ recommendations were accepted and help to optimize statin therapy prescribing [9]. Moreover, it was revealed that the pharmacist-managed program was linked to higher proportions of subjects achieving their T2DM treatment goals at three and six-month intervals in comparison with usual medical care [10].

There is well-established evidence to support achieving possible improvement in the prescribing quality through initiatives tailored for healthcare providers [11]. Also, significant differences in many prescribing elements could be achieved through educational intervention, particularly high-intensity interactive interventions [12]. In particular, academic detailing could help towards improving the prescribing quality and maintaining a needed balance between the commercial interests related to the industry and a thorough evidence-based medicine understanding related to academia [13]. Academic detailing can be described in the clinical practice as a multifaceted intervention that typically consists of a visit conducted by the qualified university-based practitioner to healthcare providers at their practice sites. The aim is to optimize the prescribing patterns in real practice through the provision of unbiased, evidence-based recommendations on best practices of drug therapies [14].

Academic detailing, a direct educational outreach intervention to promote compliance to evidence-based recommendations among healthcare providers, has been recognized as one of the effective educational interventions that help to advance the excellence of care among T2DM subjects [15]. Accordingly, there are initiatives at various levels that could help in improving the effectiveness and quality of prescribing statin medications in the current clinical practice [16]. One key initiative is the education of healthcare providers’ through the utilization of pharmacists as academic detailers which have been associated with international success stories to support rigorous, evidence-based, and cost-effective clinical practice [17]. For example, a cluster-randomized controlled trial piloted in the UK general practice to evaluate the impact of the interventions of pharmacists on enhancing the statin therapy prescribing and the achievement of cholesterol treatment goals among high CVD risk patients has revealed statistically significant improvements in the overall study outcome measures [18].

In Malaysia, national reports on the use of medicines have reported the underutilization and suboptimal use of lipid-lowering therapy in local clinical settings [19]. Also, the latest national survey among medical practitioners has highlighted gaps in both their knowledge and practice of the management of dyslipidemia [20]. Educational interventions could have a fundamental role in enlightening and promoting the effective, rational, and safe utilization of LLT among high CVD risk individuals such as T2DM subjects. Our study was aimed at designing and implementing an academic detailing program in Malaysian hospital and primary care settings and test its impact on improving the overall use of LLT e.g. statin therapy in the clinical practice.

## Materials and methods

### Study design

**Fig 1:**
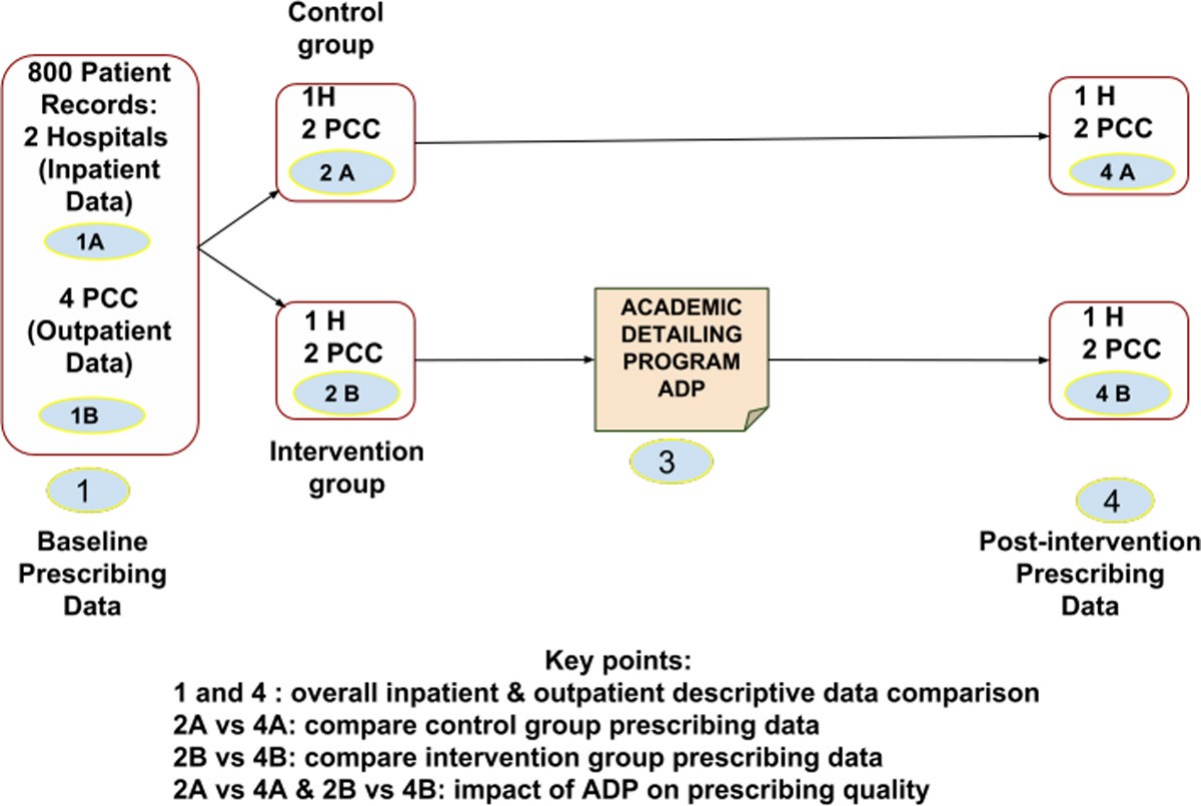
Schematic diagram of the study design.

A quasi-experimental design with a control group and pre-tests involving six healthcare practice sites in the state of Pahang, Malaysia. We conducted the first phase at all sites in the form of cross-sectional studies in order to provide baseline assessment data. Then we conducted the educational intervention program at three study sites, and the other three sites were considered as the control group. Subsequently, we conducted the post-intervention phase at all sites employing similar methods as per the baseline data collection. The assessment of the appropriate prescribing of lipid-lowering therapy for CVD prophylaxis in Malaysian patients with T2DM was based on the National CPG for the treatment of T2DM published in 2015. We evaluated and compared between the appropriate statin therapy prescribing pre and post educational intervention in all study sites. We included hospitals and primary care clinics in both intervention and control groups to provide a comprehensive assessment of the educational intervention. **Fig 1**illustrates the overall schematic study design.

### First phase: Baseline data collection

The pre-intervention data collection phase involved all eligible Malaysian patients diagnosed with T2DM from two hospitals and four primary care clinics. The data collection forms were designed to capture the patient’s demographics, proposed indication for statin therapy, and all relevant laboratory values. In addition, an overview of underlying diseases, co-prescribed medications, and description of actually or previously prescribed statin therapy were also collected. The data were collected from medical records and patients’ follow up files. The study sample was divided into two comparable matched groups; an interventional group and a control group. Each group involved one hospital and two primary care clinics. The considerations for setting comparable groups include type and number of study sites in each group, sample size from each study site and from overall sites in each group, and location of study sites.

### Second phase: Intervention

We employed a convenience sampling method to select the health care providers in one hospital and two primary care clinics (intervention group) referring to the baseline data in the first phase. An invitation to all health care providers in each facility (medical and pharmacy professionals) was undertaken to ascertain if they had been and are involved in providing care to T2DM patients. Acceptance of the invitation meant their enrolment in the intervention phase. Furthermore, the participating healthcare providers should have no recent or potential (at least three months following intervention) overlapping duties in one of the healthcare facilities in the control group.

The intervention was in the form of an academic detailing program regarding cardiovascular risk reduction with a key focus on enhancing the rational prescribing of statin therapy among Malaysian type 2 diabetic patients. The purpose of the introductory intervention component of the study was to provide an overview of the data in the first phase to provide clearer profiling of the current prescribing practice. Moreover, to help identify the potential improvement initiatives that could be considered afterward.

All core principles of academic detailing were considered [21] such as:

- Conducting surveys to investigate baseline knowledge and the motivation for current and proposed prescribing patterns. In this study, a questionnaire was developed, consisting of 12 items in order to assess the knowledge and perceptions of the current prescribing practice.
- Establishing credibility through a respected organizational sponsor and unbiased information sources. As an academic detailing program, the study was sponsored by a well-established and respected academic organization.
- Using concise and visually appealing graphical educational materials. In this study, the presentation design contained and considered recent findings regarding statin therapy use in T2DM patients. The presentation was further supported by printed materials highlighting the most recently updated evidence related to the clinical benefits and risks associated with statin therapy in T2DM patients.
- Stimulating physician participation in two-way interaction. In the educational session, the concluding component was dedicated to inciting interaction between the participating health care providers and the main researcher (the presenter). Many interesting points were discussed and voiced during this interactive discussion. For example, the physician’s experience with statin-associated muscle symptoms, the combination lipid-lowering therapy, and the indications for non-statin therapy. We have published a detailed schematic diagram of the program components and the survey used in this program [22].

### Third phase: Post-intervention

A post-intervention data collection phase from all intervention and control study sites was conducted with both subjected to intervention and those acting as control sites. From the literature review, there was no statistical difference evident between the outcome of an assessment following the latest educational outreach session at either 3 or 6 months regarding the potential change in prescribing behavior [23]. Therefore, the assessment of the impact of academic detailing on the prescribing practice was conducted at least three months following the intervention.

### Sample size calculation

The calculation was based on the equation of sample size calculation for proportion provided in the open source epidemiologic statistics for public health that suggested 369 patients’ records per study group [24]. As the study design involved two groups, one as the intervention group and the other as the control, the total expected sample size for the two groups combined was 738 in each phase. The sample size was further increased to 800 (two groups of 400 each) to address any missed or rejected cases and to facilitate the distribution of required cases between the two hospitals (200/each) and the four primary care clinics (100/each). The same sample size was anticipated to be employed for both the pre and post-intervention phases. The collection of data from the hospitals consisted of inpatient data while outpatient data were obtained from the primary care clinics to allow a comparison to be made between inpatient and outpatient lipid-lowering therapy-prescribing practice among patients with T2DM.

### Ethical approval and consent to participate

The study protocol obtained ethical approval from the Medical Research Ethical Committee (MREC) of the National Medical Research Registry (NMRR) before starting the data collection procedures. The protocol approval ID is NMRR-16-713-29691 (IRR). Further administrative approvals were also obtained in each study site upon request. All ethical rules and regulations were strictly adhered to during the research. The obtained ethical approval permit for conducting the educational intervention with all healthcare providers in the study sites mentioned above. Healthcare providers attendance and participation in our intervention imply their verbal consent to take part in our study. As per the obtained approval, there was no need for the obtainment of written consent from healthcare providers who voluntarily accepted our invitation to participate in our work.

### Inclusion criteria

The inclusion criteria incorporated data from the records of T2DM patients over the age of 40 years with or without overt CVD. Notably, all T2DM patients aged 40 to 75 years of age should receive statin therapy with the aim of CVD prevention regardless of baseline LDL cholesterol levels [25]. Furthermore, at the time of this study, participants were considered to be eligible if they were an outpatient referred to one of the four primary care clinics specified earlier; and an inpatient admitted to hospital (one of the two hospitals specified earlier) in a general medical ward for not less than 48 hours.

### Exclusion criteria

We excluded the records of T2DM patients based on the following criteria:

➢ Elderly diabetic patients > 75 years.
➢ Hospitalized patients who spent less than 48 hours in the hospital or those admitted to critical care units because those patients might be experiencing many other active medical and metabolic abnormalities requiring deterring or temporary discontinuation of statin therapy.
➢ Patients that had any contraindication to receiving statin therapy such as:
  - Known active liver diseases.
  - End-stage renal failure on dialysis with no prior history of receiving statin therapy.
  - History of statin intolerance, which did not improve with prior attempts to change statin therapy, dosage, and frequency of administration.

### Outcome measures

The primary outcome of this study aimed to identify and compare the proportion of patients on appropriate statin therapy prescribing between intervention and control groups with particular interest to examine the impact of study educational intervention. The evaluation of the appropriateness of statin therapy prescribing was mainly based on the 2015 Malaysian CPG for the treatment of patients with T2DM; recommending statin therapy for all patients between 40 and 75 years of age. The output of the evaluation process was classified into three main classifications, which were considered appropriate, inappropriate, or potentially inappropriate. The working definitions of these three classifications of statin therapy assessment are depicted in **Table 1**.

**Table 1:** Definitions of the assessment of statin therapy prescribing.

Assessment Classification	Definition
**Appropriate**	**Recommended intensity statin regimen (moderate or high intensity) for patients with high CVD risk and no potential statin-drug interaction**
**Inappropriate**	**No Statin Therapy Prescriptions**
	**Non-Statin Therapy in Statin-eligible patients**
**Potentially Inappropriate**	**Potential Statin-drug interactions**
	**Low-intensity statin in high CVD risk patients**
	**Renal dose adjustment is required for the prescribed statin regimen**

### Statistical analysis

All completed data collection sheets were included in the data analysis. Descriptive statistics using frequencies and proportions were used to describe the prevalence and evaluation of statin therapy prescribing among Malaysian patients with T2DM. Also, the Chi-square test of homogeneity was performed to compare the prevalence of statin therapy prescribing across the different study sites before and after the intervention, and the level of significance was set at P < 0.05. Data were analyzed using the Statistical Package for the Social Sciences (SPSS v.22 software. (IBM SPSS Statistics, NC, USA).

## Results

### Patients’ distribution in study sites and the demographic characteristics

In total, 782 records were examined in the hospital setting; 393 for the pre-intervention and 389 for the post-intervention phase. A total of 816 records were reviewed in the primary care setting; 404 for the pre-intervention phase and 412 for the post-intervention phase. The mean age of the study participants was 60.26 ± 8.55 and 59.05 ± 8.44 in the hospital and the primary care setting, respectively. More than half of the study sample gender was female, and the majority of patients were of Malay ethnicity. Concerning the comorbidities tracked in the study population, about 22.6% of the study subjects had an underlying atherosclerotic cardiovascular disease (ASCVD). **Table 2**displays an overview of the distribution of patients’ distribution and study sites, including their demographic characteristics and underlying diseases.

**Table 2:** Study sites distribution, demographic characteristics, and main underlying diseases among the study subjects.

	Frequency (N)	Percent (%)
**Study site (s)**		
**Hospital 1**	195	12.2
Hospital 2	198	12.4
Hospital 1*	191	12.0
Hospital 2*	198	12.4
PCC 1	104	6.6
PCC 2	99	6.2
PCC 3	100	6.2
PCC 4	101	6.3
PCC 1*	100	6.2
PCC 2*	100	6.2
PCC 3*	108	6.8
PCC 4*	104	6.5
**Gender**		
Male	693	43.4
Female	905	56.6
**Race**		
Malay	1,350	84.5
Chinese	141	8.8
Indian	94	5.9
Others	13	0.8
**ASCVD**		
Yes	361	22.6
No	1,237	77.4
**Hypertension**		
Hypertensive	1309	82
Not Hypertensive	289	18
**Dyslipidaemia**		
Yes	465	29
No	1,133	71
Total	1,598	100.0

Note: * refer to data related to the post-intervention phase.

### Patterns of statin therapy prescriptions

A considerable portion of subjects was receiving moderate intensity simvastatin therapy. Also, 2.6% of the study subjects were prescribed with combination LLT therapy, and only 6.4% were initiated on high-intensity statin therapy. **Table 3**provides a summary of the description of the prescribing of LLT for patients with T2DM in all study sites.

**Table 3:** Summary of the prescribed LLT and their relative intensities among T2DM subjects.

	Frequency (N)	Percent (%)
**Status of the Prescribed LLT**		
No LLT	322	20.2
Simvastatin	1,084	67.8
Atorvastatin	120	7.5
Other Statin Monotherapy	7	0.4
Statin Combination Therapy	41	2.6
Non-statin Monotherapy	24	1.5
**The intensity of the Prescribed Statin therapy**		
Low intensity	144	9
Moderate Intensity	1005	62.9
High Intensity	103	6.4
No Statin Therapy	346	21.7
	1,598	100.0

### The impact of academic detailing on the prescribing practice in the interventional study sites

The data from all study sites targeted by the intervention were grouped as both pre and post-intervention phases of the data assessment phase. A sample of 399 cases from the pre-intervention phase and 399 cases from the post-intervention phase were considered for analysis. A Chi-square test of homogeneity was run noting that the two multinomial probability distributions were not equal in the population, *Χ*^2^ (2) = 18.390, *p* < 0.001. However, there was a statistically, significant difference between the two proportions. The observed frequencies and percentages of the outcomes of the LLT prescribing evaluation between the two phases of data collection are presented in **Table 4**.

**Table 4:** Pre and post-intervention evaluation of LLT prescribing in all intervention study sites.

Evaluation of LLT regimen		Study sites (Intervention)		Total
		Pre Intervention	Post Intervention	
**Appropriate**	Count	188_a_	246_b_	434
	% within Study site	47.1%	61.7%	54.4%
**Potentially Inappropriate**	Count	86_a_	72_a_	158
	% within Study site	21.6%	18.0%	19.8%
**Inappropriate**	Count	125_a_	81_b_	206
	% within Study site	31.3%	20.3%	25.8%
**Total**	Count	399	399	798
	% within Study site	100.0%	100.0%	100.0%

Each subscript letter denotes a subset of the study site categories whose column proportions do not differ significantly from each other at the 0.05 level.

**Fig 2:**
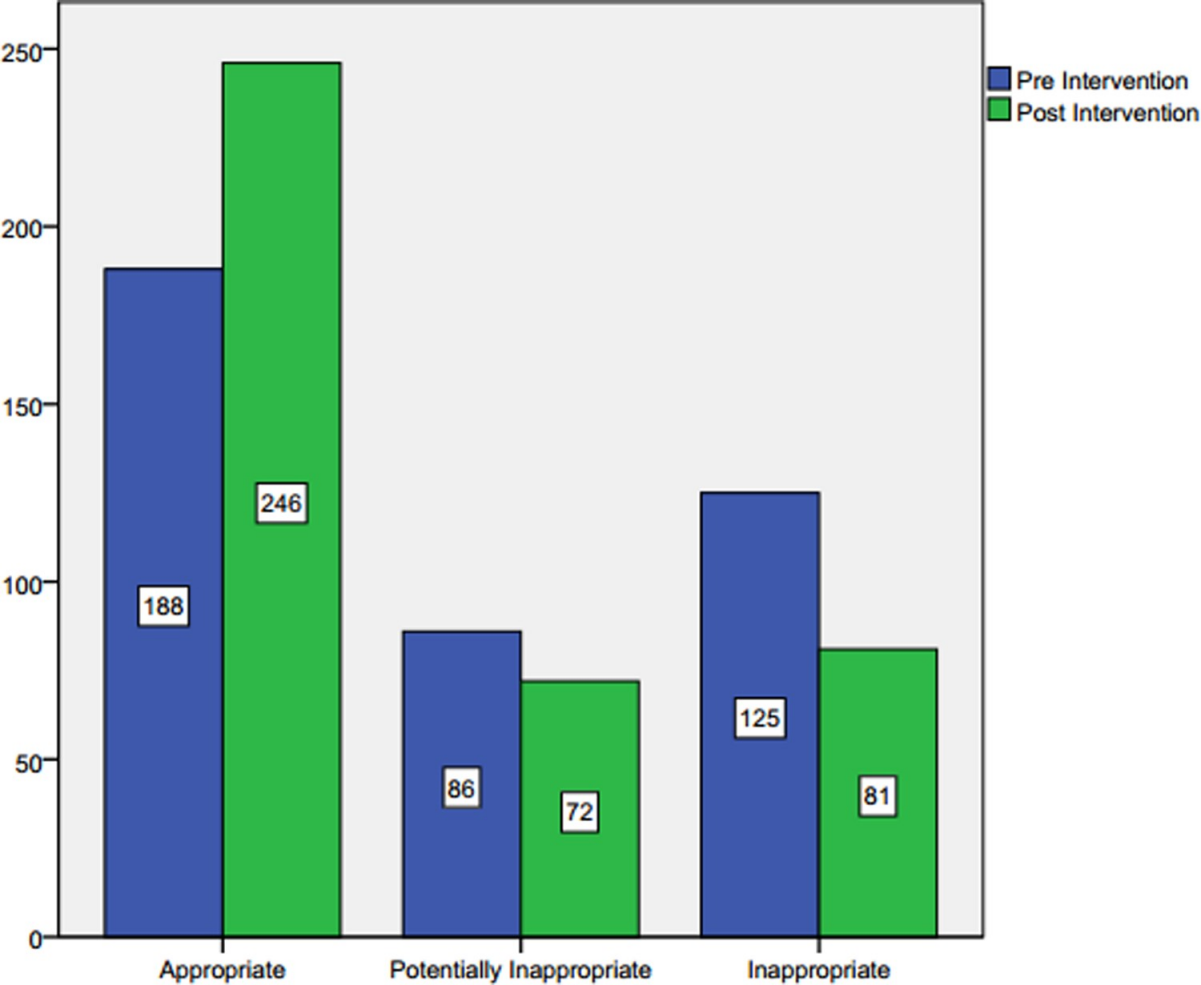
Evaluation of LLT prescribing before and after the academic detailing in the intervention study sites.

Post hoc analysis involved pairwise comparisons using multiple z-tests of two proportions with a Bonferroni correction. As we have three pairwise comparisons in the post hoc analysis, statistical significance was accepted at p < 0.0166. There was a statistically significant difference in the proportion of appropriate LLT prescribing between the post-intervention phases compared to the pre-intervention phase (n = 246, 61.7% versus n = 188, 47.1%), p = 0.001. Moreover, there was a statistically, significant difference in the proportion of inappropriate LLT prescribing between the post-intervention phases compared to the pre-intervention phase (n = 81, 20.3% versus n = 125, 31.3%). No other pairwise comparisons were associated with statistically significant differences. **Fig 2**shows the frequency differences in each category of prescribing evaluation in the intervention study sites.

### The overall impact of academic detailing on the prescribing practice in the control study sites

The assessment of LLT prescribing in the control study sites was considered as per the study design to produce a more rigorous estimation of the impact of the educational intervention. A sample of 398 cases from the pre-intervention phase and 402 cases from the post-intervention phase were considered for the analysis. A Chi-square test of homogeneity was run where the two multinomial probability distributions were not equal in the population, *Χ*^2^ (2) = 3.031, *p* = 0.220. There was no statistically, significant difference between the two proportions. The observed frequencies and percentages of the outcomes of the LLT prescribing evaluation between the two phases of data collection are presented in **Table 5**.

**Table 5:** Pre and post-intervention evaluation of LLT prescribing in all control study sites.

Evaluation of LLT regimen		Study site		Total
		Control Pre Intervention	Control Post Intervention	
**Appropriate**	Count	214	228	442
	% within Study site	53.8%	56.7%	55.3%
**Potentially Inappropriate**	Count	105	113	218
	% within Study site	26.4%	28.1%	27.3%
**Inappropriate**	Count	79	61	140
	% within Study site	19.8%	15.2%	17.5%
**Total**	Count	398	402	800
	% within Study site	100.0%	100.0%	100.0%

**Fig 3:**
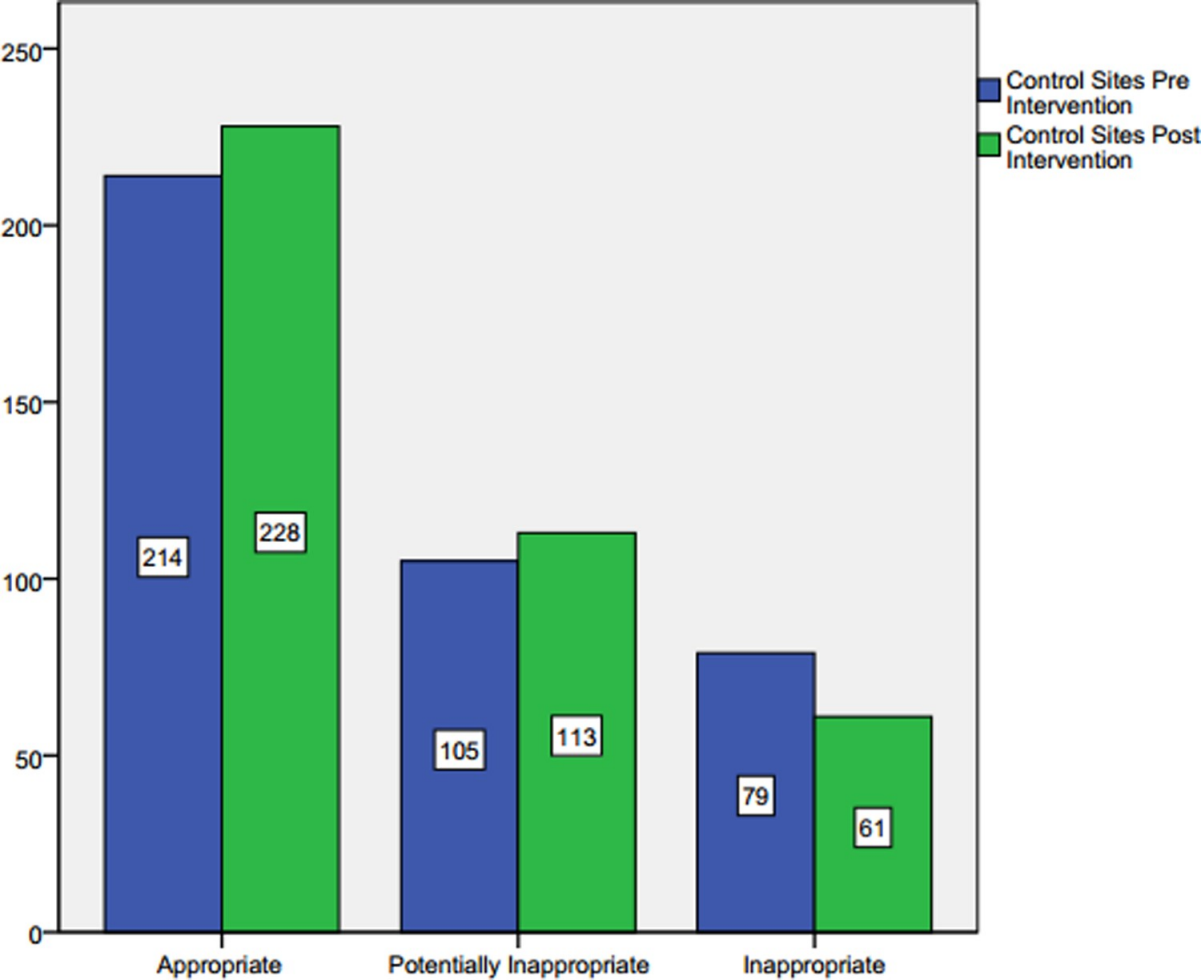
Evaluation of LLT prescribing before and after the academic detailing in the control study sites.

There was an increase in the appropriate LLT prescribing and a decrease in the inappropriate LLT prescribing. However, the magnitude of these differences was relatively small in order to show any statistical significance. **Fig 3**displays the frequency differences in each category of prescribing evaluation in the control study sites.

## Discussion

According to the results, it is shown that there is a statistically significant difference in the proportions of appropriate statin therapy prescribing between the intervention and control groups. The educational intervention in the form of the academic detailing program was found to have a positive impact on the promotion of the quality of evidence-based patient care. Similarly, the results of a US study showed that a significant gap in the evidence-based statin therapy prescribing among patients with T2DM could be filled through brief pharmacist intervention targeting healthcare providers [26]. In comparison with our study, the US study proposed an intervention designed as a phone call and fax template, while our study proposed intervention in the form of an academic detailing program involving direct interaction with the healthcare providers with relatively larger sample size.

Moreover, our intervention results are consistent with the previously reported statistically significant findings of a study that evaluated the impact of the pharmacist-led intervention on improving the achievement of cholesterol treatment goals and statin therapy prescribing among patients with high CVD risk in the UK primary care setting [18]. Compared to our study, both studies shared the same objectives of enhancing statin therapy prescribing although, in our study, the achievement of treatment goals as one of the evaluation outcomes for the intervention was not considered. Furthermore, the positive impact of our intervention in this study is consistent with the reported positive impact of the pharmacist-managed program on increasing the percentage of T2DM patients who achieved treatment goals compared with patients treated with usual care [10]. Furthermore, a positive impact was also reported following cardiovascular educational intervention targeted at healthcare professionals in the primary care setting [27].

On the other hand, a study aimed to assess the impact of the pharmacists’ intervention on increasing the guideline-directed statin therapy use among patients with acute coronary syndrome did not show a significant impact of the intervention on the overall percentage of patients receiving statin therapy [28]. This finding contradicts the results of our study, where the intervention offered by academic-affiliated pharmacists’ showed a significantly positive impact on the overall percentage of patients receiving statin therapy.

To summarise, the academic detailing program in our study showed a positive impact on statin therapy prescribing with a statistically significant difference in the percentage of the overall appropriate prescribing in both the hospital and primary care setting. Similar positive findings were frequently reported in the literature. In contrast, some studies showed the modest academic detailing impact that was not associated with statistically significant differences in the overall prescribing. Furthermore, there were some noted differences between a few studies regarding the final design of the academic detailing program, number of academic detailers, the presence of the follow-up period, and the sample size of healthcare providers and patients. In future, it is proposed to investigate the impact of these highlighted differences on the overall efficiency of the program particularly in the settings, which were not associated with satisfactory prescribing outcomes following the implementation of an academic detailing program.

## Limitations

According to the quasi-experimental design that our study applied, it would be difficult to claim that our findings could be generalized to the Malaysian T2DM population. The majority of study subjects were of Malay ethnicity; this may limit the representativeness of the findings, especially in locations where non-Malay subjects represent the majority. Moreover, we did compare both groups once after the educational intervention, so we disclose that lack of different time-based assessment at different points is a limitation of our work. Although previous research did not support major differences in the prescribing assessment at different time points [23].

## Conclusion

Our study reinforced the effect of prescribing-improvement interventions focussing on healthcare professionals who could potentially contribute towards the optimization of medications for CVD prophylaxis among T2DM patients. Also, following the comparison of the appropriate statin therapy prescribing before and after the educational intervention in both intervention and control groups, the academic detailing program was found to be significantly associated with a positive impact on the overall statin therapy prescribing among patients with T2DM. Accordingly, these results may be considered for further testing on a larger scale in the Malaysian clinical setting to promote evidence-based statin therapy use.

## Supporting information

S1 File Data collection form used throughout the research project. (PDF)

S1 Data Minimal data set from hospitals. (SAV)

S2 Data Minimal data set from primary care clinics. (SAV)

S3 Data Minimal data set for evaluation purpose. (SAV)

## Abbreviations

T2DM: type 2 diabetes mellitus
LLT: lipid-lowering therapy
CVD: cardiovascular disease
CPG: clinical practice guidelines
LDL-C: low-density lipoprotein cholesterol
H: hospital
PCC: primary care clinic
