# Supplementary material for: Pharmacist-led academic detailing improves statin therapy prescribing for Malaysian patients with type 2 diabetes: Quasi-experimental design
Source: PLoS One. 2019 Sep 19;14(9):e0220458. doi: 10.1371/journal.pone.0220458 (PMC6752830; doi:10.1371/journal.pone.0220458)
Supplement: S1 File — (PDF) [file pone.0220458.s001.pdf]

Research Project: Assessment of Prescribing Pattern of Statins in Malaysian Type 2 Diabetic Patients Pre vs. Post Educational Intervention

Data Collection Form

Study Site:	Date:	Patient's ID:

A. Patient Characteristics:

Age:	Sex :	Race :	Weight & height:
Tobacco use:<br>○ Smoker<br>○ Non-smoker<br>○ Ex-smoker		DM Onset:<br>○ 5 years and below<br>○ 5 to 10 years<br>○ More than 10 years	

B. Indication For Treatment:

Indication	
○ Type 2 DM Only (1^{ry} Prophylaxis)	○ Type 2 DM & ASCVD (2nd Prophylaxis)

C. Laboratory Investigations

Lab Investigations				
**Type**	**Value**		**Reading**	**Date**
LDL-C (mmol/L)	1^{ry} Prophylaxis	2nd Prophylaxis		
	○ < / = 2.6<br>○ > 2.6	○ < / = 1.8<br>○ > 1.8		
HDL-C (mmol/L)	Male	Female		
	○ > 1<br>○ < 1	○ > 1.2<br>○ < 1.2		
TG (mmol/L)	○ < 1.7	○ > 1.7		
Venous plasma glucose (mmol/L)	Fasting<br>○ 4.4–6.1 mmol/L<br>○ > 6.1 mmol/L	Non-fasting<br>○ 4.4–8.0 mmol/L<br>○ > 8.0 mmol/L		
AI c (%)	○ < / = 6.5%	○ > 6.5%		
Renal parameters	Creatinine value<br>(44-133 mmol/L)	Creatinine clearance<br>○ > 30 ml/min<br>○ < 30 ml/min		

Liver enzymes (U/L)	AST: (< 72 U/L)	ALT: (< 45 U/L)		

D. Drug Regimen: (statins/lipid lowering therapy)

Drug:		Dose:<br>○ 10 mg<br>○ 20 mg<br>○ 40 mg		Duration:<br>○ 1 – 2 months<br>○ 3 – 6 months<br>○ 1 year or above
Treatment Modifications types				
○ Dose intensification		○ Dose reduction		○ Discontinuation
○ Accretion		○ Subtraction		○ Switch from / to statin
If any:			Date:	

E. Concomitant Diseases/ Medical History:

Disease	Onset	Remarks
ASCVD:<br>○ CAD (coronary artery disease)<br>○ Stroke<br>○ PAD (peripheral artery disease)		
Hypertension		
Muscle Disorders		

F. Concurrent Use Of Other Medications:

Medications For Diabetes		Other Medications	
**<u>Insulin</u>**	Dose	Drug	Dose
**Oral Anti-diabetic agents**			
